# Association between Health-Related Quality of Life and Nutritional Status in Adult Patients with Crohn’s Disease

**DOI:** 10.3390/nu12030746

**Published:** 2020-03-11

**Authors:** Iolanda Cioffi, Nicola Imperatore, Olivia Di Vincenzo, Lidia Santarpia, Antonio Rispo, Maurizio Marra, Anna Testa, Franco Contaldo, Fabiana Castiglione, Fabrizio Pasanisi

**Affiliations:** 1 Internal Medicine and Clinical Nutrition Unit, Department of Clinical Medicine and Surgery, Federico II University Hospital, via Pansini 5, 80131 Naples, Italy; 2 Gastroenterology Unit, Department of Clinical Medicine and Surgery, Federico II University Hospital, via Pansini 5, 80131 Naples, Italy

**Keywords:** Quality of life, muscle strength, nutritional assessment, inflammatory bowel disease

## Abstract

This study aimed to assess health related quality of life (HRQoL) in adult patients with Crohn’s disease (CD), considering disease severity and gender differences, and also its relationship with nutritional status. Consecutive adult patients aged 18–65 years with CD were recruited. Disease activity was clinically defined by the Crohn’s Disease Activity Index (CDAI) in active and quiescent phases. HRQoL was evaluated using the validated short form (SF)-36 questionnaire for the Italian population. Additionally, anthropometry, bioimpedance analysis, and handgrip-strength (HGS) were performed. Findings showed that 135 patients (79 men and 56 women) were included, having a mean age of 38.8 ± 14 years and a BMI of 23.2 ± 3.7 kg/m^2^. Overall, active CD patients had a lower perception of their QoL compared to those clinically quiescent, while gender differences emerged mostly in the quiescent group. Interestingly, HRQoL was significantly associated with many nutritional variables, and muscle strength was the main predictor. Therefore, HRQoL is perceived lower in active compared to quiescent patients, but women experienced poorer QoL than men, especially in the quiescent phase. Finally, higher QoL scores were found in subjects being in clinical remission phase with a preserved muscle function. However, further studies are still required to verify these findings.

## 1. Introduction

Inflammatory bowel diseases (IBDs), of which Crohn’s disease (CD) is a subtype, are chronic inflammatory conditions of the gastrointestinal tract with unpredictable periods of relapse and remission [1,2,3]. Due to disease symptoms such as abdominal pain, fever, diarrhea, anorexia, and anemia, as well as complications such as surgery, abscesses, fistulas, and stenosis, CD patients are vulnerable to experiencing psychosocial issues because of changes in many aspects of social and work life, which impacts their quality of life (QoL) and wellbeing [4,5,6].

Nowadays, it is believed that the assessment of health-related QoL (HRQoL) is an important indicator of patient-reported outcomes [4,5]. Indeed, poorer social and interpersonal functioning, self-perception, and self-esteem are likely to be associated with CD-related complications such as chronic changes in bowel function, surgical scars, and ostomy, which in turn can adversely impact QoL. Findings from recent systematic reviews show that perceived QoL was significantly lower in CD patients than in healthy individuals [4,6]. In addition, within CD subjects, a lower QoL perception in the presence of active disease compared to the quiescent state has been reported, highlighting a higher impact on mental functioning [5]. However, current data are unable to examine gender differences on QoL due to the lack of detailed information.

Thus far, several studies have shown a close relationship between malnutrition and decreased quality of life in different populations [7,8,9,10,11,12,13,14], including subjects with IBD [15,16,17]. Malnutrition is frequently observed among patients with CD [18,19], and it is known to be associated with low muscle mass and an impaired functional status [15,17,20,21]. Recently, we have observed reduced muscle strength in CD patients compared to healthy controls, independent of disease activity and gender [21].

To the best of our knowledge, studies investigating factors influencing quality of life in CD patients have not yet looked at the impact of both nutritional and functional status on perceived QoL. Therefore, the objectives of the present study were: (1) to assess HRQoL in a cohort of CD patients, taking disease severity, gender differences, and previous surgery into account; and (2) to explore the relationship between HRQoL and nutritional risk.

## 2. Materials and Methods

### 2.1. Design and Study Population

This cross-sectional study explored the association between HRQoL and nutritional status in patients with CD. Participants were consecutively recruited at the department of Clinical Medicine and Surgery, Federico II University Hospital, Naples (Italy) from July 2016 until March 2018; and all detailed procedures have been already described elsewhere [21].

Patients with a diagnosis of CD aged between 18 and 65 years were included. Exclusion criteria were: history of acute or chronic liver or kidney disease, current enteral, by tube feeding, and parenteral nutrition; presence of fistulae, ileostomy, or colostomy; corticosteroids use in the last 3 months; presence of extensive small bowel resections (residual small bowel <2 m); pregnancy or lactation; rapidly changes in body weight in the last month; unable or unwilling to give informed consent.

Data on QoL derived by the short form (SF)-36 validation group for the Italian population [22] have been used as control group.

As previously reported [21], disease severity was clinically classified by the Crohn’s Disease Activity Index (CDAI), dividing patients into the active and quiescent phases (≥150 and <150, respectively). Demographic data, smoking habits, disease duration, previous surgery, drugs treatment, as well as location and disease behavior based on Montreal classification were collected. All subjects gave their informed consent before the enrollment. The study was conducted in accordance with the Declaration of Helsinki, and the protocol was approved by the Ethics Committee of the Federico II Ethical Committee (Protocol’s number:102/16) and registered in 2017 at clinicaltrials.gov as NCT03054935.

### 2.2. Health-Related Quality of Life

The validated Short-Form 36 health survey (SF-36) was employed for assessing HRQoL. The questionnaire is a generic measure of QoL, is self-administered, and consists of 36 items investigating eight dimensions or sub-scales: physical functioning, physical role, bodily pain, general health, vitality, social function, emotional role, and mental health. Scores for each dimension range from 0–100, with higher scores indicating better QoL. Two summary scores describe physical health and mental health. We used the Italian translation of the SF-36, which has been validated in Italy (IQOLA SF-36 Italian version 1.6).

### 2.3. Anthropometry, Bioimpedance Analysis and Handgrip Strength Measures

Anthropometric measures and bioimpedance analysis (BIA) were performed after an overnight fast. Body weight and height were measured to the nearest 0.1 kg and 0.5 cm, respectively, by a platform beam scale with a built-in stadiometer (Seca 709, Seca, Hamburg, Germany), while subjects wore light clothes and no shoes. BMI was calculated as body weight expressed as kilograms divided by squared height reported in meters.

BIA [23] was performed at 50 kHz using a Human Im-Touch analyzer (© DS Medica S.r.l., Milan, Italy). Measurements were carried out on the non-dominant side of the body, at room temperature between 23–25 °C and after being in the supine position for at least 10 min. The measured BIA variables were resistance (R) and reactance (Xc) [24]. Phase angle (PhA) was calculated as the ratio of R over Xc and expressed as degree, while bioimpedance index (BI-index) as squared height, reported in centimeter, divided by R (cm^2^/Ω), as detailed elsewhere [21].

Finally, handgrip strength (HGS) was measured with the dynamometer (JAMAR, Sileby, United Kingdom). Subjects were placed standing with arms outstretched parallel to the trunk, then held the dynamometer and applied maximum strength with each hand. The measurement was repeated three times alternately on both sides (dominant and non-dominant hand) with 1 min apart to avoid fatigue. The mean value was recorded in kilograms (kg) [25].

### 2.4. Statistical Analysis

Data were analyzed using SPSS IBM Statistics software (version 22). Normal distribution was assessed by the Kolmogorov–Smirnov test or Shapiro–Wilk test as appropriate. Descriptive analyses of QoL data are shown using medians, interquartile range (IQR), and intervals range. Differences between active and quiescent groups were determined using the Mann–Whitney U test. Correlation analysis was performed using Spearman’s rank coefficient. Multiple linear regression analysis was used to identify independent variables able to affect QoL, including age, gender, disease severity (CDAI), disease duration, smoking habits, previous surgery, and some nutritional indicators such as BMI, PhA, BI-index, and muscle strength. A *p* value ≤ 0.05 was considered as significant.

## 3. Results

A total of 148 patients with CD participated in this study [21], but 13 were ruled out for the following reasons: 8 subjects did not meet the inclusion criteria and 5 did not fill the SF-36 questionnaire. Thus, 135 patients, 79 men and 56 women, were included in this analysis.

The socio-demographic and clinical characteristics of patients are summarized in Table 1.

The mean age of participant was 38.8 ± 14.2 years with an average BMI of 23.2 ± 3.7 kg/m^2^.

The majority of patients were non-smokers, while about 20% smoked. Based on CDAI, 74 patients were clinically quiescent and 61 ranged between a mild to moderate active disease. Specifically, CDAI score was slightly higher in women than in men (W = 159 ± 82; M = 127 ± 76, *p* = 0.019).

The median disease duration was 6.5 years, ranging between 0.5 and 36 years. As described before [21], diagnosis was mostly done between 17 and 40 years (67%); disease location was ileocolic in 56% of patients and characterized by a stricturing phenotype (53%). In addition, only a minority of patients (20%) suffered from perianal disease, while 53% of patients underwent surgery due to medically refractory disease or complications such as strictures, abscesses, or fistulas. The use of medications was as follows: 42% of subjects took biologic agents, 27% immunosuppressive and mesalamine treatments, while about 31% patients were out of active drugs at the visit time. This section may be divided by subheadings. It should provide a concise and precise description of the experimental results, their interpretation as well as the experimental conclusions that can be drawn.

### 3.1. Description of Perceived Quality of Life

**Figure 1:**
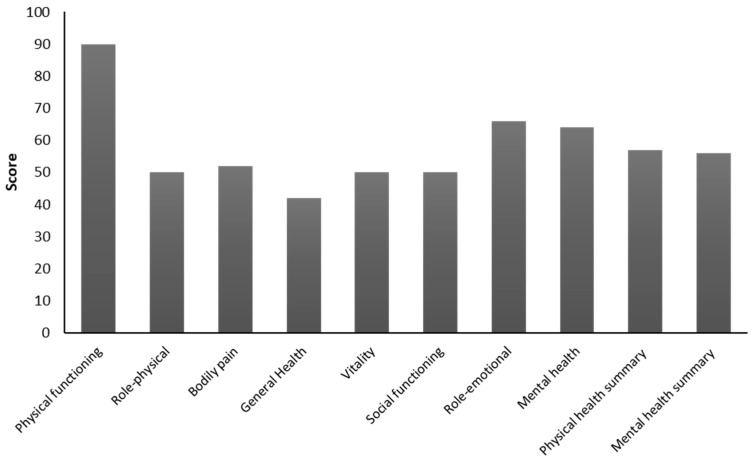
Health related quality of life in the study population.

General descriptions of eight sub-scales and the two summary scores (physical and mental health) for the study population are shown in Figure 1. The highest median value was observed for physical functioning (score = 90), while the lowest for general health perception (score = 42).

**Figure 2:**
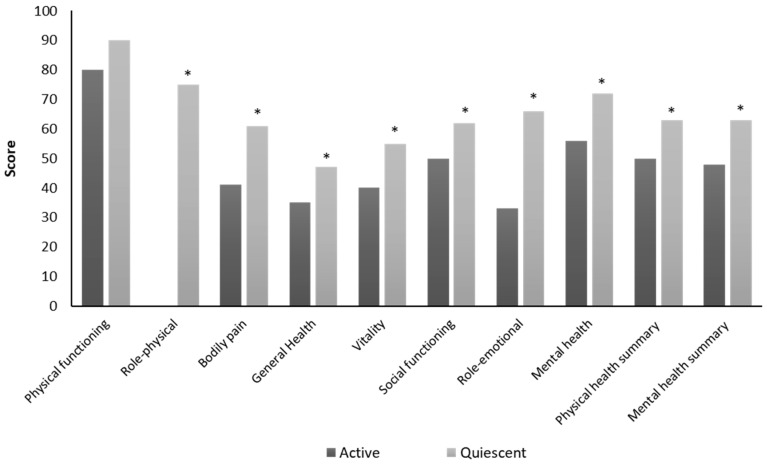
Comparison of health-related quality of life (HRQoL) between the active and the quiescent Crohn’s disease group. * *p* < 0.05

By comparing data according to CDAI, we observed an overall and significant reduction for all items in the active group compared to patients in the quiescent group (Figure 2). Interestingly, the scale related to physical functioning did not differ between the two groups (active = 80 vs. quiescent = 90; *p* = 0.13), while the role-physical dimension, which assesses the limitations in both work and daily activities due to health problems, was perceived as the lowest for the active group (median score = 0) compared to the quiescent group (median score = 75).

**Figure 3:**
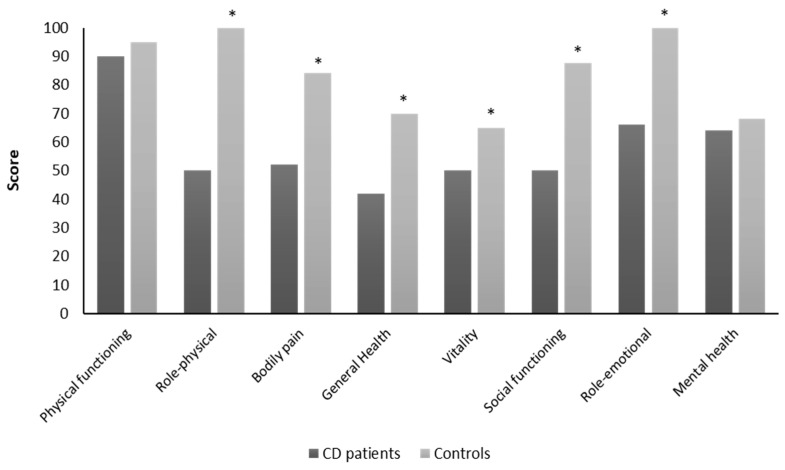
Comparison of health-related quality of life between the CD patients and controls. * *p* < 0.05.

Moreover, comparing QoL data derived from our patients with those extracted by the SF-36 validation group for the Italian population [22], all dimensions were reduced in CD patients compared to controls, except for physical functioning (Figure 3).

### 3.2. Quality of Life According to Gender and Disease Activity

When data on QoL were analyzed by gender, independent of disease activity, some differences were observed as reported in Table 2. Overall, in the whole study population, differences between genders emerged for all QoL domains. However, we did not find any significant differences between genders in the active group, even though a general reduction of all QoL items was observed. On the contrary, physical functioning, role-physical, bodily pain, general health, vitality, and mental health were significantly decreased for women compared to men (*p* < 0.05) in the quiescent group. Analyzing QoL data according to disease activity group, we observed that role-physical, general health, vitality, social functioning, role-emotional, and mental health subscales were significantly lower in male active CD patients compared to those in quiescent phase. Specifically, clinically active men perceived the physical components as very bad, especially the role physical dimension (active = 0 vs. quiescent = 100; *p* = 0.002). Likewise, all items related to the mental area such as social functioning, role emotional and mental health as well as bodily pain significantly differed among women, showing the lowest values for the active group compared to the quiescent group. Women in the active group perceived as the worst for both role-physical (active = 0 vs. quiescent = 25; *p* = 0.28) and role emotional (active = 0 vs. quiescent = 66; *p* = 0.02) compared to those in the quiescent group. However, no difference was observed for physical functioning, role-physical, general health, and vitality based on disease activity group.

### 3.3. Effect of Previous Surgery on QoL Perceptions

Nearly half of our patients had surgery due to medically refractory disease or disease complications; therefore, we examined whether QoL scores were affected by previous surgery. As reported in Table 3, role-physical, general health, and the physical health summary scores were significantly reduced in patients with surgery than those without surgery, whereas no difference was observed for the mental health scores. Interestingly, similar results were found in men, but not in women, who again showed low scores, except for physical functioning. Moreover, disease activity, assessed by CDAI, was similar between patients with and without surgery in both genders.

### 3.4. Correlation Between Nutritional Parameters and Quality of Life

As mentioned before, we have previously assessed and described bioelectrical PhA and HGS in this sample, both of which resulted in valid indicators of nutritional status [21]. In the present study, nutritional parameters were correlated with both physical and mental health summary scores using the Spearman’s linear correlation and adopting gender as controlling factor (Table 4). Findings showed that the physical health summary score was inversely correlated with age (r = −0.244; *p* = 0.005) and CDAI (r = −0.343; *p* = 0.000), whereas it was directly correlated with weight (r = 0.181; *p* = 0.038), PhA (r = 0.207; *p* = 0.017), and HGS (r = 0.333; *p* = 0.000). The mental health score was negatively associated with CDAI (*r* = −0.306; *p* = 0.000) and positively associated with all nutritional variables, but not with age (r = −0.129; *p* = 0.141).

### 3.5. Factors Influencing Quality of Life

As further step, a multiple regression analysis was performed to determine the influence of different factors, including nutritional and functional variables, on the HRQoL of patients with CD, considering the two summary scores of SF-36 as the dependent variable.

When the physical health score was set as a dependent variable, regression analysis showed that disease severity (represented by disease activity; β = -0.234, *p* = 0.004), age (β = -0.214, *p* = 0.008), and gender (β = 0.246, *p* = 0.002) were the strongest determinants of physical activity score. Similarly, by taking the mental health summary score as a dependent variable, disease severity (represented by disease activity; β = -0.279, *p* = 0.001) and gender (β = 0.257, *p* = 0.002) were the main determinants of mental QoL.

Interestingly, the inclusion of nutritional variables in the analysis showed that mean HGS was the main predictor of both physical and mental health summary scores (β = 0.392, *p* = 0.000; β = 0.395, *p* = 0.000; respectively), followed by disease severity (β = -0.253, *p* = 0.001; β = -0.308, *p* = 0.000); while age was included as predictor for the physical summary score only (β = -0.199, *p* = 0.009). Thus, higher QoL scores using the SF-36 questionnaire for both physical and mental health would typically be found in subjects being in the clinical remission phase with a preserved muscle function, even though physical health would be better perceived in younger CD patients.

## 4. Discussion

This cross-sectional study assessed HRQoL in consecutively recruited patients with CD and explored its association with nutritional status. Our findings show that QoL was strongly influenced by disease activity, with significant differences between genders in clinically quiescent patients, whereas those in the active phase had an overall low level of QoL domains. Interestingly, the presence of previous surgery due to disease complications significantly decreased general health as well as physical health summary scores in men, but not in women. With respect to the link with nutritional status, we found significant associations between QoL and functional and nutritional variables. Specifically, muscle strength was the main determinant of HRQoL in these patients, followed by disease activity, thus supporting the crucial contribution of nutritional status in affecting HRQoL.

Nowadays, HRQoL questions have become an important component of public health surveillance and are considered valid future indicators of unmet needs and intervention outcomes; hence, the assessment of QoL is crucial when deciding on optimal therapy. In the present study, we found that most of QoL domains, especially self-reported general health, were negatively impacted by the disease compared with scores obtained by the Italian population [22]. Accordingly, a recent systematic review confirmed that QoL for individuals with IBD was poorer compared to healthy individuals [4]. In addition, among IBD patients, QoL scores were even lower in patients with CD than those with ulcerative colitis, unrelated to the use of generic or specific inflammatory bowel disease questionnaire (IBDQ) [5].

Due to the relapsing course of CD, HRQoL is usually impaired at some point in every CD patient, and many of them tend to live chronically with many limitations in both work and social routine activities [6], experiencing psychological distress even in clinical remission. In this study, we found that most HRQoL domains were higher in men than in women with quiescent disease, but scores were reduced to an equally low level in both genders when disease was active, even though clinical activity of included patients ranged from mild to moderate degree.

Generally, women experience more stress in their lives than men, likely related to their different gender social role stereotypes. Previously, Sarid et al. [26] have shown no differences between genders in active phase of CD, as we found in this study; but women with quiescent disease reported greater use of emotion-focused, problem-focused, and dysfunctional coping strategies than men [27]. Accordingly, QoL scores considerably improved with reducing of disease activity in men. Instead, women in the quiescent group had a better perception, albeit lower than men, of social-functioning, role-emotional, and mental health compared to women in the active group; while no difference was observed for most scales measuring physical health in women that remained low, except for physical functioning. Based on these and previous findings, disease severity is still the main responsible factor for worsening both physical and mental HRQoL [4,5,6]. Nevertheless, disease activity contributed to only about 30% of HRQoL [28], therefore there is a need for additional, possibly modifiable, determinants of QoL among CD patients. Generally, a poor nutritional status is known to be associated with impaired functional status [29,30], worsened immune system [31], delayed recovery, higher mortality and morbidity [32], as well as with a poorer QoL.

To date, however, only a small number of studies [15,16,17] have looked at the relationship between QoL and malnutrition risk in the IBD population, with uncertain results due to confounding factors such as disease subtype, clinical activity, small sample size etc. Previously, Valentini et al. [17] reported that QoL, assessed by the German version of the Inflammatory Bowel Disease Questionnaire (IBDQ), was associated with disease activity but not with malnutrition risk or body composition variables in a cohort of 144 clinically quiescent IBD patients. Similarly, a recent study by Pulley et al. [16] showed that malnourished Australian IBD patients, screened by subjective global assessment (SGA) test, had a lower QoL scores, but neither disease activity nor SGA score was significantly correlated with QoL in their multivariate regression model. Conversely, a study by Norman et al. [15] showed that a small group of malnourished patients with IBD, still identified by a screening tool, exhibited both lower muscle mass and muscle function, suggesting that malnutrition had a measurable impact at least on the physical aspect of quality of life, which in turn affect the mental dimension.

Surprisingly, in this study, we observed that disease activity and nutritional parameters, such as body weight, BMI, phase angle, and HGS, were significantly associated with both physical and mental summary scores. The inclusion of nutritional variables in the multivariate regression model showed that muscle strength, assessed by HGS, was the strongest predictor of HRQoL, contributing for almost 40% of prediction, followed by disease activity. Therefore, higher HGS values can predict a better perception of both physical and mental summary score in these patients.

Among factors influencing QoL, we also considered the impact of previous surgery, because patients who have had surgery also experience impaired QoL [33], and disease duration, since it has been suggested that QoL may improve over time [4,5]. Overall, our results showed that patients underwent surgery felt a lower general health perception compared to those free of surgery, with similar results in men, but not in women. These data, however, are in contrast with those published by a previous study by Ponsioen et al. [34] who reported the beneficial effects of surgery compared to infliximab therapy on HRQoL in patients with limited, non-stricturing, ileocecal CD who have not responded to conventional therapy [34]. However, the effect of surgery on HRQoL was not a primary aim of the present study, therefore our data should be cautiously interpreted. Finally, regarding the role of disease duration, this work as well as most cross-sectional studies did not find any relationship between QoL and duration, likely due to the study design.

The major strengths of our study are the presentation of QoL data separated by disease activity and gender, a large sample size, and the assessment of different nutritional parameters in relation to HRQoL. While the use of a generic, not specific, QoL questionnaire, although validated in various settings [35,36,37], and the adoption of CDAI, as index for defying disease activity, could be considered as study limitations.

## 5. Conclusions

In conclusion, QoL is strongly influenced by disease activity, although the presence of previous surgery, above all in men, might be a further element that affects QoL. Overall, gender differences should be considered in the treatment of CD, as women experience major limitations in subjective well-being and daily activities than men, especially in the quiescent phase. Finally, a better nutritional status is significantly associated with higher QoL scores, resulting muscle strength the main predictor of QoL. However, further studies are needed to explore factors able to affect HRQoL in CD patients.

## Figures and Tables

**Table 1:** Demographic and clinical characteristics of patients.

Patient’s Characteristics	All
***N***	135
**Gender (Males/Females)**	79/56
**Smoking status, *n* (%)**	
Yes	26 (19.3)
No/Ex-smoker	109 (80.7)
**Clinical activity, *n* (%)**	
CDAI < 150	74 (54.8)
> 150 CDAI < 450	61 (45.2)
**Disease duration, years, median (range)**	6.5 (0.5–36)
**Montreal Classification:**	
**Age at diagnosis (A), *n* (%)**	
A1: <16 y	25 (18.5)
A2: 17–40 y	90 (66.7)
A3: >40 y	20 (14.8)
**Location (L), n (%)**	
L1: Ileum	45 (33.3)
L2: Colon	11 (8.1)
L3: Ileum and colon	76 (56.3)
L4: Upper GI tract	3 (2.2)
**Behaviour (B), *n* (%)**	
B1: Inflammatory	37 (27.4)
B2: Stricturing	72 (53.3)
B3: Penetrating	26 (19.3)
**Perianal disease, *n* (%)**	27 (20)
**Previous surgery, *n* (%)**	72 (53.3)
**Medications, *n* (%)**	
None	42 (31.1)
5-ASA	20 (14.8)
IMMs	17 (12.6)
Biologics	56 (41.5)

Crohn Disease Activity Index (CDAI); amino salicylic acid (ASA); Immunosuppressives (IMMs).

**Table 2:** Assessment of quality of life according to gender and disease activity.

	Active (M = 32; W = 29)			Quiescent (M = 47; W = 27)				All (M = 79; W = 56)		
	Median (IQR)	Range	*p* ^1^	Median (IQR)	Range	*p* ^1^	*p* ^2^	Median (IQR)	Range	*p* ^1^
Physical functioning										
Men	85 (34)	20–100	0.44	95 (20)	5–100	0.03	*0.12*	90 (30)	5–100	0.04
Women	75 (33)	15–100		80 (30)	40–100		*0.72*	80 (30)	15–100	
Role-physical										
Men	0 (94)	0–100	0.45	100 (50)	0–100	0.006	*0.002*	75 (100)	0–100	0.003
Women	0 (50)	0–100		25 (100)	0–100		*0.28*	0 (75)	0–100	
Bodily pain										
Men	52 (66)	0–100	0.14	70 (59)	0–100	0.04	*0.08*	61 (59)	0–100	0.005
Women	41 (35)	12–100		52 (33)	22–100		*0.04*	41 (33)	12–100	
General Health										
Men	40 (36)	5–86	0.21	52 (35)	10–97	0.002	*0.04*	52 (31)	5–97	0.000
Women	35 (20)	10–92		35 (27)	10–76		*0.86*	35 (22)	10–92	
Vitality										
Men	45 (34)	25–100	0.21	65 (25)	5–95	0.000	*0.003*	60 (30)	5–100	0.000
Women	40 (28)	5–75		40 (20)	10–80		*0.26*	40 (25)	5–80	
Social functioning										
Men	50 (35)	25–100	0.45	62 (50)	12–100	0.16	*0.05*	50 (50)	12–100	0.01
Women	50 (31)	0–100		62 (25)	12–100		*0.009*	50 (25)	0–100	
Role-emotional										
Men	50 (100)	0–100	0.21	100 (67)	0–100	0.12	*0.06*	66 (100)	0–100	0.01
Women	0 (66)	0–100		66 (67)	0–100		*0.02*	33 (66)	0–100	
Mental Health										
Men	56 (36)	12–100	0.16	80 (28)	16–100	0.008	*0.03*	76 (32)	12–100	0.003
Women	56 (28)	17–92		64 (20)	24–92		*0.04*	56 (20)	8–92	
Physical Health Summary										
Men	51 (50)	15–97	0.26	78 (33)	22–99	0.003	*0.01*	68 (44)	15–99	0.002
Women	38 (25)	17–92		50 (38)	21–91		*0.17*	40 (32)	17–92	
Mental Health Summary										
Men	51 (41)	19–100	0.03	72 (38)	10–98	0.008	*0.02*	67 (45)	10–100	0.001
Women	38 (35)	9–84		57 (31)	26–86		*0.01*	43 (34)	9–86	

Differences between medians were determined using the Mann–Whitney U test. IQR = interquartile range. *p*^1^ indicate differences between genders; *p*^2^ indicate differences between active and quiescent groups.

**Table 3:** Effect of previous surgery on quality of life according to gender.

	Men			Women			All		
	No Surgery (*n* = 39)	Surgery (*n* = 40)	*p*	No Surgery (*n* = 24)	Surgery (*n* = 32)	*p*	No Surgery (*n* = 63)	Surgery (*n* = 72)	*p*
Physical functioning	90 (25)	90 (34)	0.67	85 (29)	75 (34)	0.59	90 (30)	85 (30)	0.44
Role-physical	100 (75)	37.5 (100)	0.02	12.5 (75)	0 (69)	0.71	75 (100)	25 (94)	0.03
Bodily pain	74 (59)	52 (55)	0.37	41 (40)	41.5 (41)	0.73	61 (68)	52 (42)	0.32
General Health	56 (39)	42 (36)	0.004	30 (27)	37.5 (20)	0.51	47 (40)	41 (27)	0.04
Vitality	65 (25)	55 (39)	0.13	40 (31)	40 (25)	0.74	50 (35)	50 (30)	0.21
Social functioning	62 (50)	50 (47)	0.07	50 (34)	50 (22)	0.89	62 (37)	50 (35)	0.16
Role-emotional	100 (100)	49.5 (100)	0.22	33 (66)	33 (100)	0.94	66 (100)	33 (100)	0.31
Mental Health	76 (32)	74 (32)	0.55	62 (20)	56 (23)	0.16	68 (32)	60 (32)	0.18
Physical Health Summary	77.5 (30)	58.9 (51)	0.04	47.8 (29)	37.4 (37)	0.53	64.5 (44)	51.5 (44)	0.04
Mental Health Summary	73 (41)	63.7 (46)	0.12	46.2 (32)	43 (36)	0.89	60 (42)	53.2 (39)	0.15

Data are expressed as medina and interquartile range (IQR). Differences between medians were determined using the Mann–Whitney U test.

**Table 4:** Spearman’s correlation coefficients between quality of life (QoL) and nutritional variables.

	Physical Components	Mental Components
Age, years	−0.244 **	−0.129
CDAI	−0.343 **	−0.306 **
Weight, kg	0.181 *	0.262 **
BMI, kg/m^2^	0.104	0.213 *
Phase angle, °	0.207 *	0.201 *
BI-index, cm^2^/Ω	0.061	0.103
HGS, kg	0.333 **	0.321 **

Data were controlled for gender. CDAI: Chron’s Disease Activity Index; BMI = body mass index; BI-index: bio-impedance index; HGS: handgrip strength. * *p* < 0.05; ** *p* < 0.01.
